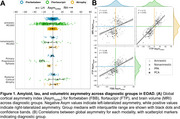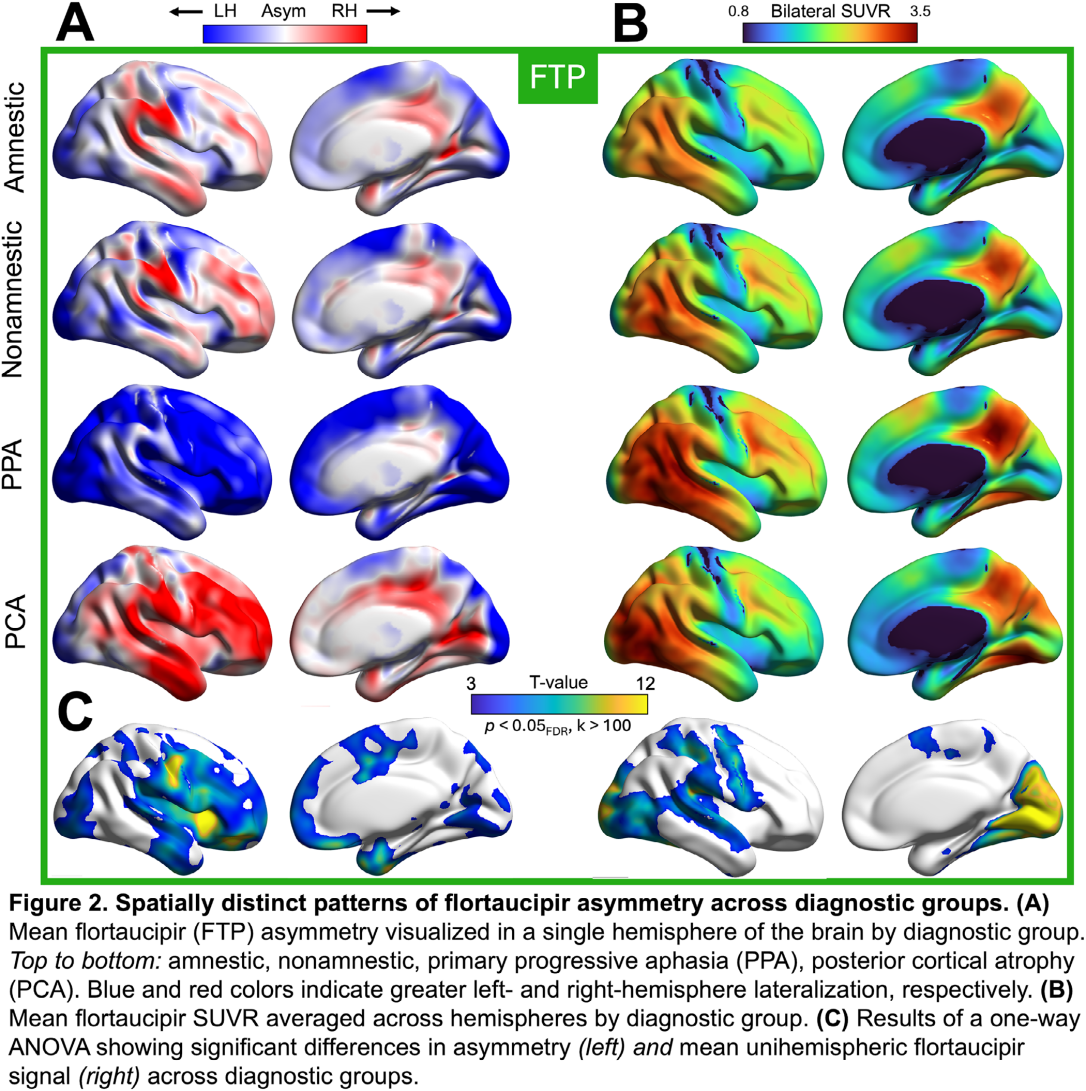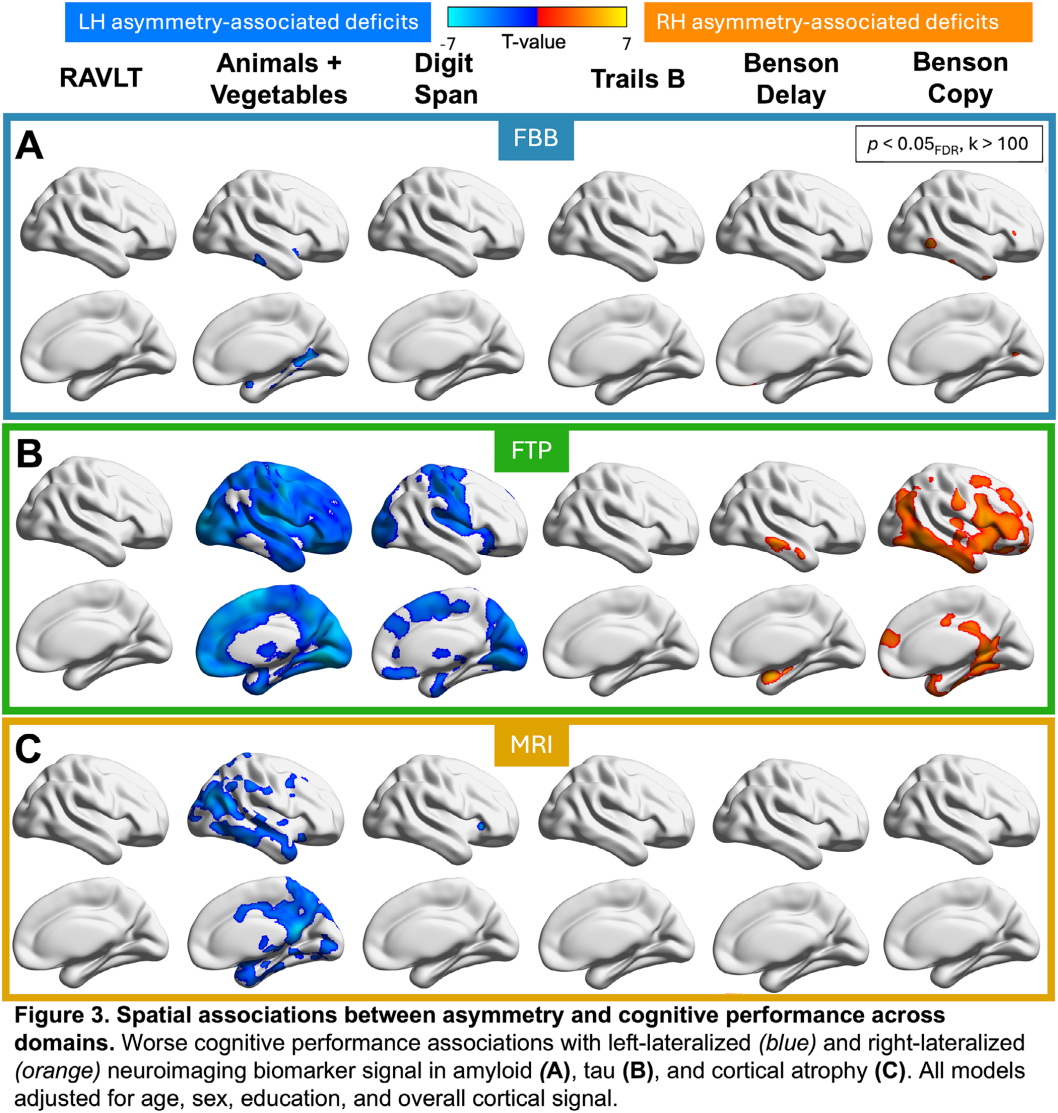# Clinical and cognitive manifestations of hemispheric asymmetry in neuroimaging biomarkers in early onset AD

**DOI:** 10.1002/alz70857_101192

**Published:** 2025-12-24

**Authors:** Jacob Ziontz, Piyush Maiti, Jiaxiuxiu Zhang, Konstantinos Chiotis, Ganna Blazhenets, Salma Rocha, Ranjani Shankar, Alinda Amuiri, Daniel R. Schonhaut, Dustin B. Hammers, Ani Eloyan, Robert A. Koeppe, Brad Dickerson, Liana G. Apostolova, Gil D. Rabinovici, Renaud La Joie

**Affiliations:** ^1^ Memory and Aging Center, Weill Institute for Neurosciences, University of California, San Francisco, San Francisco, CA, USA; ^2^ Memory and Aging Center, Weill Institute for Neurosciences, University of California San Francisco, San Francisco, CA, USA; ^3^ Indiana University School of Medicine, Indianapolis, IN, USA; ^4^ Department of Biostatistics, Brown University, Providence, RI, USA; ^5^ University of Michigan, Ann Arbor, MI, USA; ^6^ Department of Neurology, Massachusetts General Hospital and Harvard Medical School, Boston, MA, USA; ^7^ Memory and Aging Center, UCSF Weill Institute for Neurosciences, University of California, San Francisco, San Francisco, CA, USA

## Abstract

**Background:**

Asymmetrical imaging findings have been observed in individuals across the AD continuum. However, the prevalence and consequences of asymmetry in clinically heterogeneous early‐onset AD (EOAD) is not well understood.

**Method:**

We included 373 amyloid‐PET‐positive patients under 65 years with MCI or mild dementia from the Longitudinal Early Onset Alzheimer's Disease Study. Patient clinical phenotypes were amnestic or nonamnestic EOAD, primary progressive aphasia (PPA), or posterior cortical atrophy (PCA). Participants had florbetaben and flortaucipir PET, MRI, and neuropsychological testing at baseline. For each imaging modality, we used Freesurfer7.1 segmentation to compute a global asymmetry index: left/right hemisphere signal difference in all cortical regions divided by bilateral signal. To examine spatial patterns of asymmetry, we used a symmetrical, sample‐specific template to calculate an analogous asymmetry value in each PET/MRI voxel in a single hemisphere of the brain.

**Result:**

Global asymmetry was observed across modalities, most prominently in flortaucipir (Figure 1). Both left and right asymmetry was evident in all clinical phenotypes, though flortaucipir signal was markedly left‐lateralized for PPA right‐lateralized for PCA. Asymmetry was correlated between modalities, particularly flortaucipir and atrophy. Voxelwise analyses showed heterogeneous patterns of flortaucipir asymmetry in amnestic and nonamnestic groups (Figure 2). Left‐lateralized signal was prominent across cortex except in tempoparietal regions in PPA, and right‐lateralized signal was prominent except in occipital regions in PCA. For all clinical phenotypes, asymmetry was minimal in areas of highest flortaucipir signal (averaged across hemispheres): one‐way ANOVA revealed nonoverlapping regions of group differences in asymmetry and average signal. Adjusting for age, sex, education, and overall cortical abnormality in the whole sample, flortaucipir asymmetry was most robustly associated with worse cognitive performance, followed by atrophy and florbetaben (Figure 3). Worse verbal fluency and working memory related to left‐lateralized flortaucipir signal in frontal and occipital cortex. Worse performance in visuospatial tasks was associated with right‐lateralized signal in frontotemporal areas; right‐predominant medial temporal asymmetry correlated with worse delayed figure recall performance.

**Conclusion:**

Asymmetric neuroimaging patterns are common in EOAD and correlate with clinical presentation. Specific early cognitive deficits may be predicted by examining patterns of asymmetry with heterogeneous clinical and cognitive manifestations.